# Chebyshev series: Derivation and evaluation

**DOI:** 10.1371/journal.pone.0282703

**Published:** 2023-03-09

**Authors:** Robert Reynolds, Allan Stauffer

**Affiliations:** Department of Mathematics and Statistics, York University, Toronto, ON, Canada; Federal University of Technology - Parana, BRAZIL

## Abstract

In this paper we use a contour integral method to derive a bilateral generating function in the form of a double series involving Chebyshev polynomials expressed in terms of the incomplete gamma function. Generating functions for the Chebyshev polynomial are also derived and summarized. Special cases are evaluated in terms of composite forms of both Chebyshev polynomials and the incomplete gamma function.

## 1 Introduction

### 1.1 Theoretical background

If *H*(*x*, *y*, *t*) can be expanded in powers of *t* in the form

$$\begin{array}{c} H\left(x,y,t\right)=\sum_{n=0}^{\infty }h_{n}f_{n}\left(x\right)g_{n}\left(y\right)t^{n}, \end{array}$$ (1)

where *h*_*n*_ is independent of *x* and *y*, and *f*_*n*_(*x*) and *g*_*n*_(*x*) are different functions, we utilise the language employed by Rainville see p.170 in [1] and call *H*(*x*, *y*, *t*) a bilateral generating function. A double Chebyshev series is one that has two univariate Chebyshev polynomials *T*_*n*,*p*_(*α*, *β*) = *T*_*n*_(*α*)*T*_*p*_(*β*) as products in each term of the series, where *T*_*n*_(*α*) = cos(*nθ*) and *α* = cos(*θ*). A contour integral method used by Brafman [2] was used to obtain generating functions satisfying a Rodigues-type formula reducible to the form

$$\begin{array}{c} f_{n}\left(x\right)=\frac{1}{n!}D_{x}^{n}[{\left(ax+b\right)}^{n}F\left(x\right)], \end{array}$$ (2)

where *a* and *b* are constants, not both zero and *F*(*x*) is independent of *n* and differentiable an arbitrary number of times.

### 1.2 Recent developments

Mathematicians around the world have remained interested in generating functions from 1965 to present. Publications from India, Japan, Canada, the United States, England, Russia, Germany, and other European nations provide proof of this interest. Three excellent works on special functions were published in 1968 alone. Each of these took into account the idea of the generating function from a group-theoretic perspective. They were authored by American Willard Miller, Jr. [3], Canadian James D. Talman [4], and Russian N.J. Vilenkin [5]. (V. N. Singh translated Vileqkin’s novel into English). Publications on generating functions by N. A. AI-Salam and W. A. AI-Salam [6], J. W. Brown [7], and L. Carlitz [8] are only a few of the many intriguing ones along with more recent interesting publications. The Special Functions Section of the Mathematical Reviews contains a wealth of references to publications on generating functions. Literature on Chebyshev polynomials are presented in great detail in section 18 in [9]. These polynomials have well known generating functions in current literature and are used widely in all areas of mathematics and science. A particular popular application of these generating functions is in the solution of partial differential equations. Famous mathematicians like Lanczos [10] used the bivariate form of these generating functions along with their strong convergence properties in solving ordinary differential equations. In the work by Mason [11] the bivariate form of Chebyshev polynomials was employed in studying polynomial approximation. Examples of bilateral generating functions and their derivations are detailed in chapter 1 in McBride [12] and in the work by Mohammad [13]. The Chebyshev polynomial has also been studied and used in numerical solutions of initial boundary equations [14–16]. Generating functions involving special functions have been studied in the work by Meena et al. [17–20]. Solutions of partial differential equations in terms of two-dimensional Cbebyshev series have been studied with possible applications to the eigenvalue problem for a vibrating *L*-shaped membrane. In this work we provide a closed form solution to the bilateral generating function featuring the double summation of Chebyshev polynomials expressed in terms of the incomplete gamma function similar to the forms given in the works [12, 13]. In section 3, the Chebyshev contour integral formula is derived. In section 4, we give a detailed account of the incomplete gamma including integral and summation definitions. In section 5, the contour integral representations for the incomplete gamma function are derived. section 6, we formulate the main theorem and deduce a few propositions and examples in terms of constant and composite functions. In section 7, we look at the limiting case of the difference of the bivariate Chebyshev polynomials expressed in terms of the incomplete gamma function. Our preliminaries start with the contour integral method [21], applied to the formula for the Chebyshev generating function given by equation (18.12.8) in [9]. Let *a*, *α*, *β*, *k* and *w* be general complex numbers, *n* ∈ [0, ∞) and *p* ∈ [0, ∞), where the contour integral form of the Chebyshev generating function is given by

$$\begin{array}{c} \frac{1}{2\pi i}\int_{C}\sum_{n,p\geq 0}T_{n}\left(\alpha \right)T_{p}\left(\beta \right)a^{w}w^{-k+n+p-1}dw\\ =\frac{1}{2\pi i}\int_{C}\frac{a^{w}w^{-k-1}\left(1-\alpha w\right)\left(1-\beta w\right)}{\left(w^{2}-2\alpha w+1)(w^{2}-2\beta w+1\right)}dw \end{array}$$ (3)

where |*Re*(*w*)| < 1. We will use Eq (3) to derive equivalent sums for the left-hand side and a Special function form for the right-hand side. The derivation of the summation follows the method used by us in [21] which involves Cauchy’s integral formula. The generalized Cauchy’s integral formula is given by

$$\begin{array}{c} \frac{y^{k}}{\Gamma (k+1)}=\frac{1}{2\pi i}\int_{C}\frac{e^{wy}}{w^{k+1}}dw. \end{array}$$ (4)

where *C* is in general, an open contour in the complex plane where the bilinear concomitant has the same value at the end points of the contour. This method involves using a form of Eq (4) then multiply both sides by a function, then take a definite sum of both sides. This yields a definite sum in terms of a contour integral. A second contour integral is derived by multiplying Eq (4) by a function and performing some substitutions so that the contour integrals are the same.

## 2 The left-hand side contour integral

In this section we derive the infinite sum representation involving the product of two generalized Chebyshev polynomials over independent indices for the left-hand side of Eq (3). Using a generalization of Cauchy’s integral formula (4), first replace *y* → log(*a*), *k* → *k* − *n* − *p* then multiply both sides by *T*_*n*_(*α*)*T*_*p*_(*β*) and take the sums over *n* ∈ [0, ∞) and *p* ∈ [0, ∞) and simplify to get

$$\begin{array}{c} \sum_{n,p\geq 0}\frac{T_{n}\left(\alpha \right)T_{p}\left(\beta \right){\text{log}}^{k-n-p}\left(a\right)}{\Gamma (k-n-p+1)}\\ =\frac{1}{2\pi i}\sum_{n,p\geq 0}\int_{C}a^{w}T_{n}\left(\alpha \right)T_{p}\left(\beta \right)w^{-k+n+p-1}dw\\ =\frac{1}{2\pi i}\int_{C}\sum_{n,p\geq 0}a^{w}T_{n}\left(\alpha \right)T_{p}\left(\beta \right)w^{-k+n+p-1}dw\\ =\frac{1}{2\pi i}\int_{C}\frac{a^{w}w^{-k-1}\left(1-\alpha w\right)\left(1-\beta w\right)}{\left(w^{2}-2\alpha w+1)(w^{2}-2\beta w+1\right)}dw \end{array}$$ (5)

from Eq (3) where |*Re*(*w*)| < 1 and *Im*(*w*) > 0 in order for the sums to converge. Apply Tonelli’s theorem for multiple sums, see page 177 in [22] as the summands are of bounded measure over the space C×[0,∞)×[0,∞).

## 3 The incomplete gamma function

The incomplete gamma functions [9], *γ*(*a*, *z*) and Γ(*a*, *z*), are defined by

$$\begin{array}{c} \gamma \left(a,z\right)=\int_{0}^{z}t^{a-1}e^{-t}dt \end{array}$$ (6)

and

$$\begin{array}{c} \Gamma \left(a,z\right)=\int_{z}^{\infty }t^{a-1}e^{-t}dt \end{array}$$ (7)

where *Re*(*a*) > 0. The incomplete gamma function has a recurrence relation given by

$$\begin{array}{c} \gamma (a,z)+\Gamma (a,z)=\Gamma (a) \end{array}$$ (8)

where *a* ≠ 0, −1, −2, … The incomplete gamma function is continued analytically by

$$\begin{array}{c} \gamma \left(a,ze^{2m\pi i}\right)=e^{2\pi mia}\gamma \left(a,z\right) \end{array}$$ (9)

and

$$\begin{array}{c} \Gamma \left(a,ze^{2m\pi i}\right)=e^{2\pi mia}\Gamma \left(a,z\right)+\left(1-e^{2\pi mia}\right)\Gamma \left(a\right) \end{array}$$ (10)

where m∈Z, $\gamma^{*}\left(a,z\right)=\frac{z^{-a}}{\Gamma (a)}\gamma \left(a,z\right)$ is entire in *z* and *a*. When *z* ≠ 0, Γ(*a*, *z*) is an entire function of *a* and *γ*(*a*, *z*) is meromorphic with simple poles at *a* = −*n* for *n* = 0, 1, 2, … with residue $\frac{{\left(-1\right)}^{n}}{n!}$. These definitions are listed in Section 8.2(i) and (ii) in [9]. The incomplete gamma functions are particular cases of the more general hypergeometric and Meijer G functions see section (5.6) and equation (6.9.2) in [23]. Some Meijer G representations we will use in this work are given by;

$$\begin{array}{c} \Gamma \left(a,z\right)=\Gamma \left(a\right)-G_{1,2}^{1,1}(z|\begin{array}{c} 1\\ a,0 \end{array}) \end{array}$$ (11)

and

$$\begin{array}{c} \Gamma \left(a,z\right)=G_{1,2}^{2,0}(z|\begin{array}{c} 1\\ 0,a \end{array}) \end{array}$$ (12)

from equations (2.4) and (2.6a) in [24]. We will also use the derivative notation given by;

$$\begin{array}{c} \frac{\partial \Gamma (a,z)}{\partial a}=\Gamma \left(a,z\right)\text{log}\left(z\right)+G_{2,3}^{3,0}(z|\begin{array}{c} 1,1\\ 0,0,a \end{array}) \end{array}$$ (13)

from equations (2.19a) in [24], (9.31.3) in [25] and equations (5.11.1), (6.2.11.1) and (6.2.11.2) in [26], and (6.36) in [27].

## 4 Derivation of the incomplete gamma function contour integral representations

In this section we derive the general case of the Incomplete Gamma function in terms of the Cauchy contour integral. This formula will be used in the proceeding section to derive the equivalent Incomplete Gamma function contour integral representations for the right-hand side of Eq (3). Using a generalization of Cauchy’s integral formula (4), first replace *y* → *y* + log(*a*) then multiply both sides by *e*^*xy*^ and take the definite integral over *y* ∈ [0, ∞) and simplify to get

$$\begin{array}{c} \frac{a^{-x}{\left(-x\right)}^{-k-1}\Gamma \left(k+1,-x\;\text{log}\;\left(a\right)\right)}{\Gamma (k+1)}=-\frac{1}{2\pi i}\int_{C}\frac{a^{w}w^{-k-1}}{w+x}dw \end{array}$$ (14)

from equation (3.462.12) in [25] where *Re*(*x*) < 0, |arglog(*a*)| < *π*.

### 4.1 Derivation of the right-hand side contour integral representations

In this sub-section we derive the Incomplete Gamma function in terms of the contour integral representation for the right-hand side of Eq (3). These formulae are achieved by analyzing the right-hand side of Eq (3) and decomposing the quotient of polynomials into partial fractions and applying a definite integral.

#### 4.1.1 Right-hand side first contour integral

Use Eq (14) and replace $x\to -\alpha -i\sqrt{1-\alpha^{2}},k\to k+1$ and multiply both sides by $\frac{i}{4\sqrt{1-\alpha^{2}}\left(\alpha -\beta \right)}$ and simplify to get

$$\begin{array}{c} \frac{ia^{\alpha +i\sqrt{1-\alpha^{2}}}{\left(\alpha +i\sqrt{1-\alpha^{2}}\right)}^{-k-2}\Gamma (k+2,-((-\alpha -i\sqrt{1-\alpha^{2}})\text{log}\left(a\right)))}{4\sqrt{1-\alpha^{2}}\Gamma \left(k+2\right)\left(\alpha -\beta \right)}\\ =-\frac{1}{2\pi i}\int_{C}\frac{ia^{w}w^{-k-2}}{4\sqrt{1-\alpha^{2}}\left(\alpha -\beta \right)(-i\sqrt{1-\alpha^{2}}-\alpha +w)}dw \end{array}$$ (15)

#### 4.1.2 Right-hand side second contour integral

Use Eq (15) and replace *k* → *k* − 1 and multiply both sides by $-\frac{i(\alpha +\beta )}{4\sqrt{1-\alpha^{2}}\left(\alpha -\beta \right)}$ and simplify to get

$$\begin{array}{c} -\frac{ita^{\alpha +i\sqrt{1-\alpha^{2}}}{\left(\alpha +i\sqrt{1-\alpha^{2}}\right)}^{-k-1}\Gamma (k+1,-((-\alpha -i\sqrt{1-\alpha^{2}})\text{log}\left(a\right)))}{4\sqrt{1-\alpha^{2}}\Gamma \left(k+1\right)\left(\alpha -\beta \right)}\\ =\frac{1}{2\pi i}\int_{C}\frac{ita^{w}w^{-k-1}}{4\sqrt{1-\alpha^{2}}\left(\alpha -\beta \right)(-i\sqrt{1-\alpha^{2}}-\alpha +w)}dw \end{array}$$ (16)

#### 4.1.3 Right-hand side third contour integral

Use Eq (14) and replace $x\to -\alpha +i\sqrt{1-\alpha^{2}},k\to k+1$ and multiply both sides by $-\frac{i}{4\sqrt{1-\alpha^{2}}\left(\alpha -\beta \right)}$ and simplify to get

$$\begin{array}{c} -\frac{ia^{\alpha -i\sqrt{1-\alpha^{2}}}{\left(\alpha -i\sqrt{1-\alpha^{2}}\right)}^{-k-2}\Gamma (k+2,-((i\sqrt{1-\alpha^{2}}-\alpha )\text{log}\left(a\right)))}{4\sqrt{1-\alpha^{2}}\Gamma \left(k+2\right)\left(\alpha -\beta \right)}\\ =\frac{1}{2\pi i}\int_{C}\frac{ia^{w}w^{-k-2}}{4\sqrt{1-\alpha^{2}}\left(\alpha -\beta \right)(i\sqrt{1-\alpha^{2}}-\alpha +w)}dw \end{array}$$ (17)

#### 4.1.4 Right-hand side fourth contour integral

Use Eq (14) and replace $x\to -\alpha +i\sqrt{1-\alpha^{2}}$ and multiply both sides by $-\frac{i(\alpha +\beta )}{4\sqrt{1-\alpha^{2}}\left(\alpha -\beta \right)}$ and simplify to get

$$\begin{array}{c} \frac{ia^{\alpha -i\sqrt{1-\alpha^{2}}}\left(\alpha +\beta \right){\left(\alpha -i\sqrt{1-\alpha^{2}}\right)}^{-k-1}\Gamma (k+1,-((i\sqrt{1-\alpha^{2}}-\alpha )\text{log}\left(a\right)))}{4\sqrt{1-\alpha^{2}}\Gamma \left(k+1\right)\left(\alpha -\beta \right)}\\ =-\frac{1}{2\pi i}\int_{C}\frac{i\left(\alpha +\beta \right)a^{w}w^{-k-1}}{4\sqrt{1-\alpha^{2}}\left(\alpha -\beta \right)(i\sqrt{1-\alpha^{2}}-\alpha +w)}dw \end{array}$$ (18)

#### 4.1.5 Right-hand side fifth contour integral

Use Eq (14) and replace $x\to -\alpha -i\sqrt{1-\alpha^{2}},k\to k-1$ and multiply both sides by $\frac{i\alpha \beta }{4\sqrt{1-\alpha^{2}}\left(\alpha -\beta \right)}$ and simplify to get

$$\begin{array}{c} \frac{i\alpha \beta a^{\alpha +i\sqrt{1-\alpha^{2}}}{\left(\alpha +i\sqrt{1-\alpha^{2}}\right)}^{-k}\Gamma (k,-((-\alpha -i\sqrt{1-\alpha^{2}})\text{log}\left(a\right)))}{4\sqrt{1-\alpha^{2}}\Gamma \left(k\right)\left(\alpha -\beta \right)}\\ =-\frac{1}{2\pi i}\int_{C}\frac{i\alpha \beta a^{w}w^{-k}}{4\sqrt{1-\alpha^{2}}\left(\alpha -\beta \right)(-i\sqrt{1-\alpha^{2}}-\alpha +w)}dw \end{array}$$ (19)

#### 4.1.6 Right-hand side sixth contour integral

Use Eq (14) and replace $x\to -\alpha +i\sqrt{1-\alpha^{2}},k\to k-1$ and multiply both sides by $-\frac{i\alpha \beta }{4\sqrt{1-\alpha^{2}}\left(\alpha -\beta \right)}$ and simplify to get

$$\begin{array}{c} -\frac{i\alpha \beta a^{\alpha -i\sqrt{1-\alpha^{2}}}{\left(\alpha -i\sqrt{1-\alpha^{2}}\right)}^{-k}\Gamma (k,-((i\sqrt{1-\alpha^{2}}-\alpha )\text{log}\left(a\right)))}{4\sqrt{1-\alpha^{2}}\Gamma \left(k\right)\left(\alpha -\beta \right)}\\ =\frac{1}{2\pi i}\int_{C}\frac{i\alpha \beta a^{w}w^{-k}}{4\sqrt{1-\alpha^{2}}\left(\alpha -\beta \right)(i\sqrt{1-\alpha^{2}}-\alpha +w)}dw \end{array}$$ (20)

#### 4.1.7 Right-hand side seventh contour integral

Use Eq (14) and replace $x\to -\beta -i\sqrt{1-\beta^{2}},k\to k+1$ and multiply both sides by $-\frac{i}{4\sqrt{1-\beta^{2}}\left(\alpha -\beta \right)}$ and simplify to get

$$\begin{array}{c} -\frac{ia^{\beta +i\sqrt{1-\beta^{2}}}{\left(\beta +i\sqrt{1-\beta^{2}}\right)}^{-k-2}\Gamma (k+2,-((-\beta -i\sqrt{1-\beta^{2}})\text{log}\left(a\right)))}{4\sqrt{1-\beta^{2}}\Gamma \left(k+2\right)\left(\alpha -\beta \right)}\\ =\frac{1}{2\pi i}\int_{C}\frac{ia^{w}w^{-k-2}}{4\sqrt{1-\beta^{2}}\left(\alpha -\beta \right)(-i\sqrt{1-\beta^{2}}-\beta +w)}dw \end{array}$$ (21)

#### 4.1.8 Right-hand side eighth contour integral

Use Eq (14) and replace $x\to -\beta -i\sqrt{1-\beta^{2}}$ and multiply both sides by $\frac{i(\alpha +\beta )}{4\sqrt{1-\beta^{2}}\left(\alpha -\beta \right)}$ and simplify to get

$$\begin{array}{c} \frac{ita^{\beta +i\sqrt{1-\beta^{2}}}{\left(\beta +i\sqrt{1-\beta^{2}}\right)}^{-k-1}\Gamma (k+1,-((-\beta -i\sqrt{1-\beta^{2}})\text{log}\left(a\right)))}{4\sqrt{1-\beta^{2}}\Gamma \left(k+1\right)\left(\alpha -\beta \right)}\\ =-\frac{1}{2\pi i}\int_{C}\frac{ita^{w}w^{-k-1}}{4\sqrt{1-\beta^{2}}\left(\alpha -\beta \right)(-i\sqrt{1-\beta^{2}}-\beta +w)}dw \end{array}$$ (22)

#### 4.1.9 Right-hand side ninth contour integral

Use Eq (14) and replace $x\to -\beta -i\sqrt{1-\beta^{2}},k\to k-1$ and multiply both sides by $\frac{i\alpha \beta }{4\sqrt{1-\beta^{2}}\left(\alpha -\beta \right)}$ and simplify to get

$$\begin{array}{c} -\frac{i\alpha \beta a^{\beta +i\sqrt{1-\beta^{2}}}{\left(\beta +i\sqrt{1-\beta^{2}}\right)}^{-k}\Gamma (k,-((-\beta -i\sqrt{1-\beta^{2}})\text{log}\left(a\right)))}{4\sqrt{1-\beta^{2}}\Gamma \left(k\right)\left(\alpha -\beta \right)}\\ =\frac{1}{2\pi i}\int_{C}\frac{i\alpha \beta a^{w}w^{-k}}{4\sqrt{1-\beta^{2}}\left(\alpha -\beta \right)(-i\sqrt{1-\beta^{2}}-\beta +w)}dw \end{array}$$ (23)

#### 4.1.10 Right-hand side tenth contour integral

Use Eq (14) and replace $x\to -\beta +i\sqrt{1-\beta^{2}},k\to k+1$ and multiply both sides by $\frac{i}{4\sqrt{1-\beta^{2}}\left(\alpha -\beta \right)}$ and simplify to get

$$\begin{array}{c} \frac{ia^{\beta -i\sqrt{1-\beta^{2}}}{\left(\beta -i\sqrt{1-\beta^{2}}\right)}^{-k-2}\Gamma (k+2,-((i\sqrt{1-\beta^{2}}-\beta )\text{log}\left(a\right)))}{4\sqrt{1-\beta^{2}}\Gamma \left(k+2\right)\left(\alpha -\beta \right)}\\ =-\frac{1}{2\pi i}\int_{C}\frac{ia^{w}w^{-k-2}}{4\sqrt{1-\beta^{2}}\left(\alpha -\beta \right)(i\sqrt{1-\beta^{2}}-\beta +w)}dw \end{array}$$ (24)

#### 4.1.11 Right-hand side eleventh contour integral

Use Eq (14) and replace $x\to -\beta +i\sqrt{1-\beta^{2}}$ and multiply both sides by $-\frac{i(\alpha +\beta )}{4\sqrt{1-\beta^{2}}\left(\alpha -\beta \right)}$ and simplify to get

$$\begin{array}{c} -\frac{ia^{\beta -i\sqrt{1-\beta^{2}}}\left(\alpha +\beta \right){\left(\beta -i\sqrt{1-\beta^{2}}\right)}^{-k-1}\Gamma (k+1,-((i\sqrt{1-\beta^{2}}-\beta )\text{log}\left(a\right)))}{4\sqrt{1-\beta^{2}}\Gamma \left(k+1\right)\left(\alpha -\beta \right)}\\ =\frac{1}{2\pi i}\int_{C}\frac{i\left(\alpha +\beta \right)a^{w}w^{-k-1}}{4\sqrt{1-\beta^{2}}\left(\alpha -\beta \right)(i\sqrt{1-\beta^{2}}-\beta +w)}dw \end{array}$$ (25)

#### 4.1.12 Right-hand side twelfth contour integral

Use Eq (14) and replace $x\to -\beta +i\sqrt{1-\beta^{2}},k\to k-1$ and multiply both sides by $\frac{i\alpha \beta }{4\sqrt{1-\beta^{2}}\left(\alpha -\beta \right)}$ and simplify to get

$$\begin{array}{c} \frac{i\alpha \beta a^{\beta -i\sqrt{1-\beta^{2}}}{\left(\beta -i\sqrt{1-\beta^{2}}\right)}^{-k}\Gamma (k,-((i\sqrt{1-\beta^{2}}-\beta )\text{log}\left(a\right)))}{4\sqrt{1-\beta^{2}}\Gamma \left(k\right)\left(\alpha -\beta \right)}\\ =-\frac{1}{2\pi i}\int_{C}\frac{i\alpha \beta a^{w}w^{-k}}{4\sqrt{1-\beta^{2}}\left(\alpha -\beta \right)(i\sqrt{1-\beta^{2}}-\beta +w)}dw \end{array}$$ (26)

## 5 Main results

In this section we develop a theorem, propositions and examples to demonstrate the potential application of the double Chebyshev series. In this section we will use $\alpha -i\sqrt{1-\alpha^{2}}=f_{1}$, $\alpha +i\sqrt{1-\alpha^{2}}=f_{2}$, $\beta -i\sqrt{1-\beta^{2}}=f_{3}$, $\beta +i\sqrt{1-\beta^{2}}=f_{4}$, $\text{f4},\sqrt{1-\alpha^{2}}=f_{5}$ and $\sqrt{1-\beta^{2}}=f_{6}$ For all a,k,α,β∈C then,

$$\begin{array}{c} \sum_{n,p\geq 0}\frac{T_{n}\left(\alpha \right)T_{p}\left(\beta \right)}{{\left(a\pi \right)}^{n+p}{\left(k\right)}_{-n-p+1}}\\ =-\frac{1}{4f_{5}f_{6}k\left(k+1\right)\left(\alpha -\beta \right)}i\pi^{-k}a^{-k}(\alpha \beta f_{6}k\left(k+1\right)e^{\pi af_{1}}f_{1}^{-k}\Gamma \left(k,af_{1}\pi \right)\\ -\alpha f_{6}\left(k+1\right)e^{\pi af_{1}}f_{1}^{-k-1}\Gamma \left(k+1,af_{1}\pi \right)-\beta f_{6}\left(k+1\right)e^{\pi af_{1}}f_{1}^{-k-1}\Gamma \left(k+1,af_{1}\pi \right)\\ +f_{6}e^{\pi af_{1}}f_{1}^{-k-2}\Gamma \left(k+2,af_{1}\pi \right)-\alpha \beta f_{6}k\left(k+1\right)e^{\pi af_{2}}f_{2}^{-k}\Gamma \left(k,af_{2}\pi \right)\\ +\alpha f_{6}\left(k+1\right)e^{\pi af_{2}}f_{2}^{-k-1}\Gamma \left(k+1,af_{2}\pi \right)+\beta f_{6}\left(k+1\right)e^{\pi af_{2}}f_{2}^{-k-1}\Gamma \left(k+1,af_{2}\pi \right)\\ -f_{6}e^{\pi af_{2}}f_{2}^{-k-2}\Gamma \left(k+2,af_{2}\pi \right)-\alpha \beta f_{5}k\left(k+1\right)e^{\pi af_{3}}f_{3}^{-k}\Gamma \left(k,af_{3}\pi \right)\\ +\alpha f_{5}\left(k+1\right)e^{\pi af_{3}}f_{3}^{-k-1}\Gamma \left(k+1,af_{3}\pi \right)+\beta f_{5}\left(k+1\right)e^{\pi af_{3}}f_{3}^{-k-1}\Gamma \left(k+1,af_{3}\pi \right)\\ -f_{5}e^{\pi af_{3}}f_{3}^{-k-2}\Gamma \left(k+2,af_{3}\pi \right)+\alpha \beta f_{5}k\left(k+1\right)e^{\pi af_{4}}f_{4}^{-k}\Gamma \left(k,af_{4}\pi \right)\\ -\alpha f_{5}\left(k+1\right)e^{\pi af_{4}}f_{4}^{-k-1}\Gamma \left(k+1,af_{4}\pi \right)-\beta f_{5}\left(k+1\right)e^{\pi af_{4}}f_{4}^{-k-1}\Gamma \left(k+1,af_{4}\pi \right)\\ +f_{5}e^{\pi af_{4}}f_{4}^{-k-2}\Gamma \left(k+2,af_{4}\pi \right)) \end{array}$$ (27)

Observe the right-hand side of Eq (5) is equivalent to the addition of the right-hand sides of Eqs (15) to (26) then the left-hand sides are equal. We then apply Eq (3) and replace *a* → *e*^*aπ*^ and simplify the Gamma function and use the Pochhammer symbol see equation (5.2.5) in [9] to yield the stated result.

$$\begin{array}{c} \sum_{n,p\geq 0}\frac{T_{n}\left(i\right)T_{p}\left(-i\right)e^{-4(n+p)}\pi^{-n-p}}{{\left(\frac{1}{2}\right)}_{-n-p+1}}\\ =\frac{1}{8}((2+\sqrt{2})e^{-i(\sqrt{2}-1)e^{4}\pi }E_{\frac{1}{2}}(-i(-1+\sqrt{2})e^{4}\pi )+(2+\sqrt{2})e^{i(\sqrt{2}-1)e^{4}\pi }\\ E_{\frac{1}{2}}(i(-1+\sqrt{2})e^{4}\pi )-(\sqrt{2}-2)e^{-i(1+\sqrt{2})e^{4}\pi }\\ E_{\frac{1}{2}}(-i(1+\sqrt{2})e^{4}\pi )-(\sqrt{2}-2)e^{i(1+\sqrt{2})e^{4}\pi }E_{\frac{1}{2}}(i(1+\sqrt{2})e^{4}\pi )+16) \end{array}$$ (28)

Use Eq (27) and set *k* = 1/2, *a* = *e*^4^, *α* = *i*, *β* = −1 and simplify using equation (8.19.1) in [9].

$$\begin{array}{c} \sum_{n,p\geq 0}\frac{1}{{\left(a\pi \right)}^{n+p}{\left(k\right)}_{1-n-p}}=1+\frac{1}{k}+e^{a\pi }\left(1+k-a\pi \right)E_{1-k}\left(a\pi \right) \end{array}$$ (29)

Use Eq (27) and apply l’Hopital’s rule as *α* → 1, *β* → 1 and simplify using equations (18.5.14) and (8.19.1) in [9].

$$\begin{array}{c} \sum_{n,p\geq 0}\frac{{\left(-1\right)}^{n+p}({\left(2\right)}_{-1+n+p}\left(-1+\gamma +\psi^{\left(0\right)}\left(1+n+p\right)\right))}{{\left(a\pi \right)}^{n+p}}\\ =-1+e^{a\pi }E_{2}\left(a\pi \right)-e^{a\pi }G_{2,3}^{3,0}(a\pi |\begin{array}{c} 3,3\\ 1,2,2 \end{array}) \end{array}$$ (30)

Use Eq (29) take the first partial derivative with respect to *k* and set *k* = −1 and simplify using Eq (13) and the Pochhammer symbol equation given by $\frac{1}{{\left(k\right)}_{-n-p+1}}={\left(-1\right)}^{n+p+1}{\left(1-k\right)}_{n+p-1}$ where $k\in C,n,p\in N_{0}$.

$$\begin{array}{c} \sum_{n,p\geq 0}\frac{\text{cos}(n\alpha )\text{cos}(p\beta )}{{\left(a\pi \right)}^{n+p}{\left(k\right)}_{1-n-p}}\\ =\frac{1}{4ki\left(1+k\right){\left(a\pi \right)}^{k}\left(\text{cos}\left(\alpha \right)-\text{cos}\left(\beta \right)\right)}(e^{ae^{-i\alpha }\pi }{\left(e^{-i\alpha }\right)}^{-k}k\left(1+k\right)\text{cos}\left(\beta \right)\text{cot}\left(\alpha \right)\Gamma (k,ae^{-i\alpha }\pi )\\ -e^{ae^{i\alpha }\pi }{\left(e^{i\alpha }\right)}^{-k}k\left(1+k\right)\text{cos}\left(\beta \right)\text{cot}\left(\alpha \right)\Gamma (k,ae^{i\alpha }\pi )\\ -k\left(1+k\right)\text{cos}\left(\alpha \right)\text{cot}\left(\beta \right)(e^{ae^{-i\beta }\pi }{\left(e^{i\beta }\right)}^{k}\Gamma (k,ae^{-i\beta }\pi )-e^{ae^{i\beta }\pi }{\left(e^{-i\beta }\right)}^{k}\Gamma (k,ae^{i\beta }\pi ))\\ -e^{ae^{-i\alpha }\pi +i\alpha }{\left(e^{-i\alpha }\right)}^{-k}\left(1+k\right)\left(\text{cos}\left(\alpha \right)+\text{cos}\left(\beta \right)\right)\text{csc}\left(\alpha \right)\Gamma (1+k,ae^{-i\alpha }\pi )\\ +e^{ae^{i\alpha }\pi -i\alpha }{\left(e^{i\alpha }\right)}^{-k}\left(1+k\right)\left(\text{cos}\left(\alpha \right)+\text{cos}\left(\beta \right)\right)\text{csc}\left(\alpha \right)\Gamma (1+k,ae^{i\alpha }\pi )\\ +e^{ae^{-i\beta }\pi +i\beta }{\left(e^{i\beta }\right)}^{k}\left(1+k\right)\left(\text{cos}\left(\alpha \right)+\text{cos}\left(\beta \right)\right)\text{csc}\left(\beta \right)\Gamma (1+k,ae^{-i\beta }\pi )\\ -e^{ae^{i\beta }\pi -i\beta }{\left(e^{-i\beta }\right)}^{k}\left(1+k\right)\left(\text{cos}\left(\alpha \right)+\text{cos}\left(\beta \right)\right)\text{csc}\left(\beta \right)\Gamma (1+k,ae^{i\beta }\pi )\\ +e^{ae^{-i\alpha }\pi +2i\alpha }{\left(e^{-i\alpha }\right)}^{-k}\text{csc}\left(\alpha \right)\Gamma (2+k,ae^{-i\alpha }\pi )-e^{ae^{i\alpha }\pi -2i\alpha }{\left(e^{i\alpha }\right)}^{-k}\text{csc}\left(\alpha \right)\Gamma (2+k,ae^{i\alpha }\pi )\\ -e^{ae^{-i\beta }\pi +2i\beta }{\left(e^{i\beta }\right)}^{k}\text{csc}\left(\beta \right)\Gamma (2+k,ae^{-i\beta }\pi )+e^{ae^{i\beta }\pi -2i\beta }{\left(e^{-i\beta }\right)}^{k}\text{csc}\left(\beta \right)\Gamma (2+k,ae^{i\beta }\pi )) \end{array}$$ (31)

Use Eq (27) replace *α* → cos(*α*), *β* → cos(*β*) and simplify the left-hand side using equation (18.5.1) in [9]. Product of Cosine Functions in terms of the Complementary Error Function erfc(z) and error function erfc(z).

$$\begin{array}{c} \sum_{n,p\geq 0}\frac{e^{-4(n+p)}\text{cos}(\frac{\pi n}{2})\text{cos}(\frac{\pi p}{4})}{{\left(-\frac{1}{2}\right)}_{-n-p+1}}\\ =(\frac{1}{4}+\frac{i}{4})e^{2}\sqrt{\pi }((2+i\sqrt{2})e^{-ie^{4}}(\text{erf}({\left(-1\right)}^{3/4}e^{2})+1)\\ +2{\left(-1\right)}^{7/8}e^{-{\left(-1\right)}^{3/4}e^{4}}(\text{erf}({\left(-1\right)}^{7/8}e^{2})+1)\\ +2{\left(-1\right)}^{5/8}e^{\sqrt[{4}]{-1}e^{4}}\text{erfc}(\sqrt[{8}]{-1}e^{2})-(\sqrt{2}+2i)e^{ie^{4}}\text{erfc}(\sqrt[{4}]{-1}e^{2})) \end{array}$$ (32)

Use Eq (31) set *k* = −1/2, *a* = *e*^4^/*π*, *α* = *π*/2, *β* = *π*/4 and simplify the right-hand side using equations (8.4.1) and (8.4.6) in [9]. The value of (−1)^3/4^ used in the calculation is in the first quadrant which is *e*^*πi*/4^. The Golden Ratio.

$$\begin{array}{c} \sum_{n,p\geq 0}\frac{T_{n}(\sqrt{5})T_{p}(\frac{\sqrt{5}}{2})}{{\left(a\pi \right)}^{n+p}{\left(k\right)}_{1-n-p}}\\ =\frac{1}{k}+\frac{1}{4\sqrt{5}}((4+\sqrt{5})e^{(-2+\sqrt{5})a\pi }E_{1-k}((-2+\sqrt{5})a\pi )\\ +(-1+\sqrt{5})e^{\frac{1}{2}(-1+\sqrt{5})a\pi }E_{1-k}(\frac{1}{2}(-1+\sqrt{5})a\pi )\\ +(1+\sqrt{5})e^{\frac{1}{2}(1+\sqrt{5})a\pi }E_{1-k}(\frac{1}{2}(1+\sqrt{5})a\pi )\\ +(-4+\sqrt{5})e^{(2+\sqrt{5})a\pi }E_{1-k}((2+\sqrt{5})a\pi )) \end{array}$$ (33)

Use Eq (31) set $\alpha \to \sqrt{5},\beta \to \frac{\sqrt{5}}{2}$ and simplify the right-hand side using equations (8.4.1) and (8.4.6) in [9].

## 6 The limiting case of the difference of a negative index

In this section we will derive a few generating functions using the identity *T*_*n*_(−*x*) = −1^*n*^*T*_*n*_(*x*) which is listed in Table (18.6.1) in [9]. We proceed by using Eq (27) and forming a second equation by replacing *α* → *α*, *β* → −*β* taking their difference and simplifying. Next we evaluate five cases listed below when *α* = *β* = 1, *α* = *β* = 2, *α* = *β* = 3, *α* = *β* = 4, *α* = *β* = 5. In order to simplify the right-hand sides of these formulae we apply the limit and simplify. The simplification process is not very easy and tedious, however the results are very inetersting.

$$\begin{array}{c} \sum_{n,p\geq 0}\frac{-1+{\left(-1\right)}^{n+p}}{{\left(a\pi \right)}^{n+p}{\left(k\right)}_{1-n-p}}=\frac{e^{-(a+ik)\pi }}{{\left(a\pi \right)}^{k}k}(k(1+k+a\pi )\Gamma (k,-a\pi )\\ +e^{a\pi }(({\left(-a\right)}^{k}-a^{k}e^{ik\pi })\left(1+k\right)\pi^{k}-e^{(a+ik)\pi }k\left(1+k-a\pi \right)\Gamma \left(k,a\pi \right))) \end{array}$$ (34)

$$\begin{array}{c} \sum_{n,p\geq 0}\frac{(-1+{\left(-1\right)}^{n+p})T_{n}\left(2\right)T_{p}\left(2\right)}{{\left(a\pi \right)}^{n+p}{\left(k\right)}_{1-n-p}}\\ =\frac{a^{-k}e^{-((2+\sqrt{3})a+ik)\pi }}{12k}(6e^{(2+\sqrt{3})a\pi }({\left(-a\right)}^{k}-a^{k}e^{ik\pi })\left(2+k\right)\\ +k(-a^{k}e^{4a\pi +ik\pi }(6+2\sqrt{3}+3k+3(-2+\sqrt{3})a\pi )E_{1-k}(-((-2+\sqrt{3})a\pi ))\\ +{\left(-a\right)}^{k}e^{2\sqrt{3}a\pi }(6+2\sqrt{3}+3k-3(-2+\sqrt{3})a\pi )E_{1-k}((-2+\sqrt{3})a\pi )\\ +{\left(-a\right)}^{k}(6-2\sqrt{3}+3k+3(2+\sqrt{3})a\pi )E_{1-k}(-((2+\sqrt{3})a\pi ))\\ +a^{k}e^{(2(2+\sqrt{3})a+ik)\pi }(-6+2\sqrt{3}-3k+3(2+\sqrt{3})a\pi )E_{1-k}((2+\sqrt{3})a\pi ))) \end{array}$$ (35)

$$\begin{array}{c} \sum_{n,p\geq 0}\frac{(-1+{\left(-1\right)}^{n+p})T_{n}\left(3\right)T_{p}\left(3\right)}{{\left(a\pi \right)}^{n+p}{\left(k\right)}_{1-n-p}}\\ =\frac{1}{16k}{\left(-3+2\sqrt{2}\right)}^{-2k}a^{-k}{\left((-3+2\sqrt{2})a\right)}^{-k}e^{(-((3+2\sqrt{2})a)+2ik)\pi }\\ {\left(-((3+2\sqrt{2})\pi )\right)}^{-k}(8{\left(3-2\sqrt{2}\right)}^{2k}{\left(-a\right)}^{k}e^{(3+2\sqrt{2})a\pi }({\left(-a\right)}^{k}-a^{k}e^{ik\pi })\left(2+k\right)\pi^{k}\\ +{\left(3-2\sqrt{2}\right)}^{3k}{\left(-a\right)}^{k}k(8-3\sqrt{2}+4k+4(3+2\sqrt{2})a\pi )\Gamma (k,-((3+2\sqrt{2})a\pi ))\\ +{\left((-3+2\sqrt{2})a\right)}^{k}k(-e^{6a\pi +ik\pi }(8+3\sqrt{2}+4k+4(-3+2\sqrt{2})a\pi )\Gamma (k,(3-2\sqrt{2})a\pi )\\ +e^{4\sqrt{2}a\pi }((8+3\sqrt{2}+4k+4(3-2\sqrt{2})a\pi )\Gamma (k,(-3+2\sqrt{2})a\pi )\\ +{\left(3-2\sqrt{2}\right)}^{2k}e^{6a\pi +ik\pi }(-8+3\sqrt{2}-4k+4(3+2\sqrt{2})a\pi )\Gamma (k,(3+2\sqrt{2})a\pi )))) \end{array}$$ (36)

$$\begin{array}{c} \sum_{n,p\geq 0}\frac{(-1+{\left(-1\right)}^{n+p})T_{n}\left(4\right)T_{p}\left(4\right)}{{\left(a\pi \right)}^{n+p}{\left(k\right)}_{1-n-p}}\\ =\frac{a^{-k}e^{-((4+\sqrt{15})a\pi )}{\left((-4+\sqrt{15})\pi \right)}^{-k}}{60k}(30e^{(4+\sqrt{15})a\pi }({\left(-a\right)}^{k}-a^{k}e^{ik\pi })\left(2+k\right){\left((4-\sqrt{15})\pi \right)}^{k}\\ -e^{8a\pi +ik\pi }k(30+4\sqrt{15}+15k+15(-4+\sqrt{15})a\pi )\Gamma (k,-((-4+\sqrt{15})a\pi ))\\ +e^{2\sqrt{15}a\pi }k(30+4\sqrt{15}+15k-15(-4+\sqrt{15})a\pi )\Gamma (k,(-4+\sqrt{15})a\pi )\\ +{\left(31-8\sqrt{15}\right)}^{k}k((30-4\sqrt{15}+15k+15(4+\sqrt{15})a\pi )\Gamma (k,-((4+\sqrt{15})a\pi ))\\ +e^{(2(4+\sqrt{15})a+ik)\pi }(-30+4\sqrt{15}-15k+15(4+\sqrt{15})a\pi )\Gamma (k,(4+\sqrt{15})a\pi ))) \end{array}$$ (37)

$$\begin{array}{c} \sum_{n,p\geq 0}\frac{(-1+{\left(-1\right)}^{n+p})T_{n}\left(5\right)T_{p}\left(5\right)}{{\left(a\pi \right)}^{n+p}{\left(k\right)}_{1-n-p}}\\ =\frac{{\left(-5+2\sqrt{6}\right)}^{-2k}a^{-k}{\left((-5+2\sqrt{6})a\right)}^{-k}}{48k}e^{(-((5+2\sqrt{6})a)+2ik)\pi }{\left(-((5+2\sqrt{6})\pi )\right)}^{-k}\\ (24{\left(5-2\sqrt{6}\right)}^{2k}{\left(-a\right)}^{k}e^{(5+2\sqrt{6})a\pi }({\left(-a\right)}^{k}-a^{k}e^{ik\pi })\left(2+k\right)\pi^{k}+{\left(5-2\sqrt{6}\right)}^{3k}\\ {\left(-a\right)}^{k}k(24-5\sqrt{6}+12k+12(5+2\sqrt{6})a\pi )\Gamma (k,-((5+2\sqrt{6})a\pi ))\\ +{\left((-5+2\sqrt{6})a\right)}^{k}k(-e^{10a\pi +ik\pi }(24+5\sqrt{6}+12k+12(-5+2\sqrt{6})a\pi )\\ \Gamma (k,(5-2\sqrt{6})a\pi )+e^{4\sqrt{6}a\pi }((24+5\sqrt{6}+12k+12(5-2\sqrt{6})a\pi )\\ \Gamma (k,(-5+2\sqrt{6})a\pi )+{\left(5-2\sqrt{6}\right)}^{2k}e^{10a\pi +ik\pi }(-24+5\sqrt{6}-12k+12(5+2\sqrt{6})a\pi )\\ \Gamma (k,(5+2\sqrt{6})a\pi )))) \end{array}$$ (38)

## 7 Generating functions involving the Chebyshev polynomial

In this section we derive a few exponential generating functions involving the Chebyshev polynomial. The method involves simultaneous equations and ordinary differential equations.

### 7.1 Initial equations

Here we identify the equation we would like to find the new representation for given by;

$$\begin{array}{c} \sum_{n\geq 0}\frac{z^{n}T_{n}\left(x\right)}{n!}=e^{xz}\;\text{cos}(\sqrt{1-x^{2}}z) \end{array}$$ (39)

Next we increase the factorial by one and assign a general function *g*(*z*) to be solved given by;

$$\begin{array}{c} \sum_{n\geq 0}\frac{z^{n}T_{n}\left(x\right)}{(n+1)!}=g\left(z\right) \end{array}$$ (40)

Next we take the difference of Eqs (39) and (40) to get;

$$\begin{array}{c} \sum_{n\geq 0}\frac{nz^{n}T_{n}\left(x\right)}{\Gamma (n+2)}=e^{xz}\;\text{cos}(\sqrt{1-x^{2}}z)-g\left(z\right) \end{array}$$ (41)

Next we take the derivative of Eq (40) such that the left-hand side is equal to the left-hand side of Eq (41) to get;

$$\begin{array}{c} \sum_{n\geq 0}\frac{nz^{n}T_{n}\left(x\right)}{\Gamma (n+2)}=zg^{\prime }\left(z\right) \end{array}$$ (42)

Since the left-hand sides of Eqs (41) and (42) are the same we may equate the right-hand sides and solve the ordinary differential equation given by;

$$\begin{array}{c} -zg^{\prime }\left(z\right)-g\left(z\right)+e^{xz}\;\text{cos}(\sqrt{1-x^{2}}z)=0 \end{array}$$ (43)

Next we solve the differential equation for the function *g*(*z*), with independent variable *z* given by;

$$\begin{array}{c} g\left(z\right)=\frac{e^{xz}(\sqrt{1-x^{2}}\;\text{sin}(\sqrt{1-x^{2}}z)+x\;\text{cos}(\sqrt{1-x^{2}}z))}{z}+\frac{c_{1}}{z} \end{array}$$ (44)

Next we apply the initial condition of *g*(0) = 0 and simplify to get;

$$\begin{array}{c} \sum_{n\geq 0}\frac{z^{n}T_{n}\left(x\right)}{(n+1)!}=\frac{e^{xz}(\sqrt{1-x^{2}}\;\text{sin}(\sqrt{1-x^{2}}z)+x\;\text{cos}(\sqrt{1-x^{2}}z))}{z}-\frac{x}{z} \end{array}$$ (45)

### 7.2 Examples of generating functions

Repeating the method above the following Chebyshev generating functions are derived;

$$\begin{array}{c} \sum_{n\geq 0}\frac{z^{n}T_{n}\left(x\right)}{(n+2)!}=\frac{x(2\sqrt{1-x^{2}}e^{xz}\text{sin}(\sqrt{1-x^{2}}z)-z)+(2x^{2}-1)e^{xz}\;\text{cos}(\sqrt{1-x^{2}}z)}{z^{2}}\\ -\frac{2x^{2}-1}{z^{2}} \end{array}$$ (46)

$$\begin{array}{c} \sum_{n\geq 0}\frac{z^{n}T_{n}\left(x\right)}{(n+3)!}=\frac{1}{z^{3}}(-\frac{1}{2}z(4x^{2}+xz-2)+\sqrt{1-x^{2}}(4x^{2}-1)e^{xz}\;\text{sin}(\sqrt{1-x^{2}}z)\\ +x(4x^{2}-3)e^{xz}\;\text{cos}(\sqrt{1-x^{2}}z))-\frac{4x^{3}-3x}{z^{3}} \end{array}$$ (47)

$$\begin{array}{c} \sum_{n\geq 0}\frac{z^{n}T_{n}\left(x\right)}{(n+4)!}=\frac{1}{z^{4}}(4x\sqrt{1-x^{2}}(2x^{2}-1)e^{xz}\text{sin}(\sqrt{1-x^{2}}z)\\ +(8x^{4}-8x^{2}+1)e^{xz}\text{cos}(\sqrt{1-x^{2}}z)\\ -\frac{1}{6}z(24x^{3}+6x^{2}z+x(z^{2}-18)-3z))-\frac{8x^{4}-8x^{2}+1}{z^{4}} \end{array}$$ (48)

$$\begin{array}{c} \sum_{n\geq 0}\frac{z^{n}T_{n}\left(x\right)}{(n+5)!}=\frac{1}{z^{5}}(-16x^{5}+20x^{3}+e^{xz}(\sqrt{1-x^{2}}(16x^{4}-12x^{2}+1)\text{sin}(\sqrt{1-x^{2}}z)\\ +x(16x^{4}-20x^{2}+5)\text{cos}(\sqrt{1-x^{2}}z))\\ -\frac{1}{24}z(192x^{4}+48x^{3}z+8x^{2}(z^{2}-24)\\ +xz(z^{2}-36)-4z^{2}+24)-5x) \end{array}$$ (49)

## 8 Discussion

In this work we used contour integration, simultaneous equations and ordinary differential equation techniques to derive infinite sum formulae involving the Chebyshev polynomial. The mathematical techniques themselves are easy to use however when applied to this special function the evaluation was not straightforward. Some of the challenges we faced were in simplifying the incomplete gamma function representation pf the contour integral along with finding the values where the infinite sum converges. The advantage of this work lies in the closed form solutions derived using these techniques. We have been able to add new formulae to current literature which we hope will be of use to the academic community.

## 9 Conclusion

In this paper, we have presented a method for deriving generating functions involving the Chebyshev polynomial and its product over independent parameter ranges along with some interesting special cases using both contour integration and well known algebraic techniques. We plan to apply these methods to derive other generating functions involving other special functions in future work. The results presented were numerically verified for both real and imaginary and complex values of the parameters in the integrals using Mathematica by Wolfram.
